# Prevalence of Underweight, Overweight and Obesity among Adults in Urban Bissau, Western Africa

**DOI:** 10.3390/nu13124199

**Published:** 2021-11-23

**Authors:** Ruben Turé, Albertino Damasceno, Mouhammed Djicó, Nuno Lunet

**Affiliations:** 1 EPIUnit—Instituto de Saúde Pública, Universidade do Porto, Rua das Taipas, nº 135, 4050-600 Porto, Portugal; tino_7117@yahoo.com.br (A.D.); nlunet@med.up.pt (N.L.); 2 Laboratório Para a Investigação Integrativa e Translacional em Saúde Populacional (ITR), Rua das Taipas, nº 135, 4050-600 Porto, Portugal; 3 Departamento de Ciências da Saúde Pública e Forenses e Educação Médica, Faculdade de Medicina da Universidade do Porto, Alameda Pro. Hernâni Monteiro, 4200-319 Porto, Portugal; 4 Faculdade de Medicina, Universidade Eduardo Mondlane, Av. Salvador Allende 702, Maputo C.P. 254, Mozambique; 5 CCM—Comissão de Coordenação Multissetorial de Luta Contra a TB, VIH e Paludismo, Rua Marien N’Gouabi, S/N, atrás de CMI de Bissau, Bissau 861, Guinea-Bissau; djicoblama.spccm@gmail.com; 6 CNEPS—Comité Nacional de Ética em Pesquisa na Saúde, Avenida Combatente da Liberdade da Pátria, Hospital “3 de Agosto”, Bissau 1004, Guinea-Bissau

**Keywords:** overweight, obesity, abdominal, health surveys, Africa, western, Guinea-Bissau

## Abstract

Overweight and obesity affect a large proportion of the population and are important causes of death in both developed and low- and middle-income countries. In Guinea-Bissau, there are no previous population-based studies assessing this phenomenon. Therefore, we aimed to quantify the prevalence of underweight, overweight, and obesity among adults in Bissau. A stratified and cluster sample of 935 adults was assembled in 2021 and was evaluated using standardized questionnaires and anthropometric measurements, following the World Health Organization Stepwise Approach to Chronic Disease Risk Factor Surveillance. Underweight, obesity, and overweight were defined by body mass index based on the World Health Organization definitions. The prevalence of overweight and obesity was 48.7% among women and 25.0% among men. The proportion of women with abdominal obesity was 14 times higher than it was in men (35.3% vs. 2.5%). The prevalence of overweight and obesity increased with age and income. Underweight was more prevalent in the age group of 18 to 24 years (18.4% in women and 28.9% in men) and was less frequent among individuals with higher socioeconomic status. In conclusion, the prevalence of overweight and obesity is similar to the trends that are observed in many other urbanized populations in Africa and is already a major public health issue in urban Guinea-Bissau.

## 1. Introduction

Overweight and obesity affect a large proportion of the population and are important causes of death both in developed and low- and middle-income countries (LMIC) [1]. In 2016, an estimated 1900 million adults worldwide were overweight, and 650 million of them were obese [2]. High body mass index (BMI) is associated with a higher risk of diabetes, hypertension, and other chronic diseases [3] and was estimated to account for four million global deaths in 2015, most of which were due to cardiovascular diseases [4,5].

The prevalence of overweight and obesity among urban dwellers in African countries has seen a rapid increase over the past two decades [6], reflecting the demographic and nutritional transitions that are occurring in the continent [7]. This includes lifestyle modifications and the westernization of diets, which may be regarded as desirable status symbols in urban communities [8]. Over one-quarter of the adults in Africa are estimated to be overweight or obese [9].

The long-term impact of overweight and obesity is well documented. Obesity has been demonstrated to be a predisposing factor in the increased prevalence of non-communicable diseases (NCDs), such as type-2 diabetes mellitus, hypertension, cancer, and stroke, among adults in Sub-Saharan Africa (SSA) [10]. Nevertheless, the health effects of obesity are often disregarded in SSA, given the importance of competing risks that are associated with communicable diseases in these settings as well as a cultural misconception that being overweight or obese may reflect good health and high socioeconomic status [11,12]. Therefore, country-specific studies are needed to understand the burden of overweight and obesity to better guide and align policies and strategies that are aimed at curbing this public health issue on the continent.

Specifically, in Guinea-Bissau, the burden of overweight and obesity is also becoming a major public health concern. In 2013, the Global Burden of Disease (GBD) study estimated the prevalence of overweight and obesity among women and men from Guinea-Bissau at 24.2% and 16.8%, respectively [13]; however, these figures were estimated through extrapolations of data from other countries. Therefore, the present study aims to quantify the prevalence of overweight and obesity among adults in urban Bissau.

## 2. Materials and Methods

### 2.1. Setting

This study was conducted in Bissau, the capital city of Guinea-Bissau. The country has 1.9 million inhabitants, and about 54% of the population is under 15 years of age [14]. The society is marked by ethnic diversity and the coexistence of different religious groups. Bissau is the largest and more affluent city, with a population of 365 thousand inhabitants distributed over 77 km^2^ [15]. The country is characterized by development issues such as a weak economy, the poor mobilization of domestic resources, a lack of dynamism in the private sector, the deficient development of human capital, and a GDP per capita of USD 727.5 (2020) [16]. In 2010, about 70% of the population lived in moderate poverty (with USD2 US dollars or less per day), and 33% of the population lived in extreme poverty [14,16]. This poverty is more accentuated in rural areas, which have high unemployment rates. Therefore, individuals from rural regions tend to move to Bissau to seek employment and a better lifestyle. Furthermore, there is a high propensity for foods that are energy dense and poor in micronutrients, such as street food. Given that street foods are generally very affordable, they become very appealing to poor individuals who need to meet their daily nutritional needs as cheaply as possible [17,18].

### 2.2. Study Design

This is a population-based cross-sectional study of adult dwellers in Bissau. Individuals aged 18 to 69 years were recruited based on a stratified and cluster sampling frame that was defined using data from the most recent census. A total of 50 primary sampling units (PSU) out of 408 geographical clusters were randomly selected, with a similar probability within each of the eight sectors of the city (strata). All of the households in each selected cluster were listed, and 2 to 12 households per cluster were randomly selected, with a probability proportional to the number of inhabitants in the corresponding strata, corresponding to a total of 373 households. All of the dwellers aged 18 to 69 years of age in each selected household were listed (as reported by the head of the family or the dweller answering the door when the former was not present), and those who were present at the home at the time were considered eligible. The first individuals who were listed were invited, up to a maximum of three. A total of 3 participants were evaluated in 272 households, and less than three eligible individuals were available or accepted to participate in the remaining (78 and 23 households with two and one participants, respectively). A total of 18 subjects refused to participate (1.8%), and 995 were evaluated between January and March 2021, based on the World Health Organization (WHO) STEPwise approach to Surveillance (STEPS) to Chronic Disease Risk Factor manual [19].

Information on age, sex, education, and income were collected through a face-to-face interview using structured questionnaires. Anthropometric measurements were performed by trained interviewers, with participants wearing light clothing and no footwear and with a scale on a leveled floor. Body weight was measured to the nearest one kg using a mechanical scale (Camry), and height was measured to the nearest 0.1 cm in the standing position using a portable stadiometer (SECA 213). BMI was calculated as weight (kg) divided by squared height (m^2^), and further weight was categorized according to the WHO [20] as underweight (<18.5 kg/m^2^), normal (18.5 to 24.99 kg/m^2^), overweight (25.0 to 29.99 kg/m^2^), and obese (≥30 kg/m^2^). A BMI ≥25.0 kg/m^2^ denotes overweight and obesity. Waist circumference was measured once, to the nearest one cm, using a constant tension tape (SECA 201) directly over the skin or over light clothing and at the level of the midpoint between the inferior margin of the last rib and the iliac crest in the mid-axillary line. Abdominal obesity was defined according to the WHO criteria [20]: waist circumference ≥94 cm for men and ≥80 cm for women; waist-to-hip ratio (WHR) ≥ 0.85 in women and ≥0.90 in men; a waist-to-height-ratio (WTHR) of >0.50 [21]. Among the participants with a normal weight (BMI 18.5 to 24.99 kg/m^2^) who had abdominal obesity were classified as having normal-weight central obesity.

### 2.3. Statistical Analysis

From a total of 995 participants, 32 had missing anthropometric data, 20 did not report their age, and a further 28 pregnant women were excluded because anthropometric parameters vary during pregnancy, yielding a final sample of 915 individuals being available for data analysis. Descriptive data are reported as absolute frequencies and percentages and means and standard deviations, as appropriate. Regarding annual income, the conversion between the local currency (Franco Communauté Financière Africaine—XOF) and United States dollars (USD) was performed using the exchange rate XOF 1 = USD 0.0018.

All of the analyses were conducted by considering the sampling weights and adjusting them for strata and clustering at the primary sampling unit level using Stata version 15 (StataCorp, College Station, TX, USA). Each participant was assigned a sample weight that was computed by considering the probability of selection at each stage of sampling, as follows: 1/(probability of selection of the corresponding PSU * probability of selection of the corresponding household within the PSU * probability of selection of the participant within the household). These weights were further corrected for the prevalence estimates to reflect the sex and age distribution of the population in the city of Bissau, as reported in the most recent census.

### 2.4. Ethics

The study protocol was approved by the Guinea-Bissau National Ethics Committee for Health (097/CNES/INASA/2020). Written informed consent was obtained from all individuals selected for the study before the interview and exam.

## 3. Results

Approximately one-third of the study population was aged 25 to 34 years of age. Nearly one-fifth of women and just over 5% of men had no formal education. A level of education that was achieved between 7 and 12 years of age was attained by 37.5% of women and 42.3% of men, while higher education was nearly twice more common among men than women (women 15.2% vs. men: 29.7%). A total of 43.1% of women and 32.8% of men reported no income in the previous year (Table 1).

The mean BMI was 25.5 kg/m^2^ in women and 22.8 kg/m^2^ in men. The overall prevalence of obesity was 12.8% and was nearly four times higher in women than it was in men (20.6% vs. 5.5%). Underweight affected around one tenth of both women and men. The mean waist circumference was also higher in women than it was in men (84.2 vs. 80.1 cm), with the prevalence of abdominal obesity being four times higher among women (55.8% vs. 13.9%) (Table 2).

The prevalence of overweight and obesity increased with age, peaking at 45 to 54 years of age among women (70.0%) and at 55 to 69 years of age in men (56.0%). There was no clear pattern of variation of overweight and obesity with education, though the lowest prevalence was observed among individuals with lower levels of education (1 to 6 years), in both sexes. The prevalence of overweight and obesity tended to increase with income in both women and men and tended to be the highest among participants with a monthly income above USD 1899. The mean waist circumference was 84.2 cm among women and 80.1 cm in men, and this increased with age for both sexes. Waist circumference was the highest among women with no formal education and among men with the highest level of income (Table 3).

**Figure 1:**
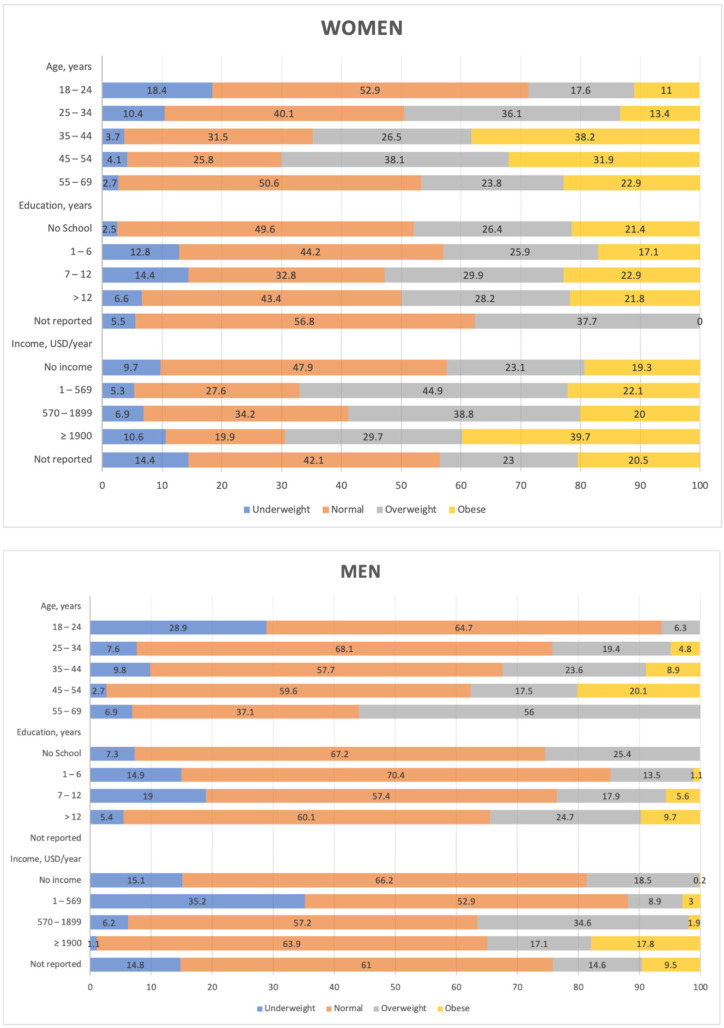
Body mass index categories by sex and socio-demographic characteristics.

As depicted in Figure 1, the prevalence of obesity among women peaked in the age group of 35 to 44 years, with a prevalence of 38.2%, and among men in the age group 45 to 54 years, with a prevalence 20.1%. The prevalence increased with education and income among men, while no substantial variation was observed with education among women, and the prevalence was higher among women with a higher income. Underweight was more prevalent in the younger age group (18 to 24 years), 18.4% in females and 28.9% in males, and tended to decrease with age. It was less frequent among men who were more educated and who had a higher income and was more frequent among women with a higher income. 

After adjusting for sex, age, education, and income, as applicable, older individuals were more likely to be overweight and obese (55–69 vs. 18–24 years, OR: 7.95, 95% CI: 3.00–21.13). Similarly, more educated individuals were more likely to be overweight or obese (>12 years vs. no school, OR: 3.00, 95% CI: 1.23–7.27) (Table 4).

The prevalence of normal-weight central obesity using waist circumference as a measure of abdominal obesity was 16.3% (32.7% among women vs. 5.9% among men), as shown in Table 5. When the waist–hip ratio and waist-to-height ratio were used to define abdominal obesity, the prevalence of normal-weight central obesity was 20.8% (25.5% among women vs. 17.7% among men) and 24.0% (30.4% among women vs. 20.0% among men), respectively. Overall, all of the measures of abdominal obesity consistently showed a higher proportion of normal-weight central obesity among women and older adults.

## 4. Discussion

This study shows that overweight and obesity affect over one-third of the adult women and nearly one-quarter of adult men in Bissau. The prevalence of overweight and obesity in the current study is similar to that reported among urban dwellers in Mozambique in 2015 (40.5% among urban women and 26.5% among urban men), Burkina Faso in 2013 (24.5% overweight and 12.5% obese), and Malawi in 2009 (23.2% overweight and 12.0% obese) [22,23,24]. Some studies in other African cities, such as Dakar (19.2% overweight and 9.7% obese) in Senegal (2015) and Ibadan (42% among women and 14% among men) in Nigeria (2010), found slightly a lower prevalence of obesity and overweight than the current study [12,25]. We speculate that this disparity might be because of differences in dietary habits and the positioning in the different phases of the demographic, nutritional, and epidemiological transition of each country [26].

There was a preponderance of obesity among women in the current study, as in many other studies, which have also indicated that overweight and/or obesity were more prevalent among women worldwide [12,22,23,27,28,29]. In SSA countries, such as Guinea-Bissau, obesity or overweight is considered to be a sign of prosperity and good health or beauty among women [30]. In contrast, being underweight is perceived as a sign of illness and poverty, and has been associated with AIDS [31]. This cultural factor in SSA women accompanied with other known factors, such as high socioeconomic status and lower levels of physical activity, leads women to be more affected by high BMI [32].

The higher prevalence of obesity among individuals with a higher income level observed in this study is consistent with other studies in LMIC [33,34]. Individuals with higher income levels in Africa tend to shift to diets that are rich in fats and oils and sugar as well as animal based products that are rich in saturated fats (diets commonly referred to as “Western Diets”) and are far less active [35]. Another finding from this study was the lack of apparent difference in the prevalence of overweight and obesity among individuals of different educational levels. If educational attainment was to be used as a proxy for the socioeconomic status of the participants, then our findings revealed that changes in the proportion of overweight and/or obese individuals are similar between the poorest and richest segments of the studied population, suggesting that poor urban dwellers are becoming as affected as their richer counterparts. This finding is in accordance with reports of an increase in prevalence of overweight and/or obesity with rapid urbanization within Africa, which is primarily driven by rural–urban migration [36]. The new migrants with low socioeconomic status tend to adapt to the urban lifestyles that predispose them to becoming overweight or obese [37].

Waist circumference can easily be applied in clinical practice [38], but the validated cut-off points more commonly utilized in screening for abdominal obesity in the world are based on a population of predominantly European origin [39]. Considering the heterogeneity of human body fat distribution across racial groups [40,41,42], in the absence of ethnic or country specific cut-off points used to define abdominal obesity, discussing the prevalence of abdominal obesity in this study needs to be approached with caution. When compared to other SSA urban areas, the mean waist circumference in Bissau, using the WHO cut-off points for increased risk of metabolic conditions, is higher than those reported in Nigeria [43], particularly among women over 40 years of age. The difference in the prevalence of abdominal obesity between women and men can be attributed to numerous factors including sex hormones that cause differences in body structure and composition [44], environmental and genetic susceptibility for the accumulation of fat [45], postpartum or postmenopausal changes in the redistribution of body fat in the abdominal area [46,47], and types of daily activities and cultural differences between genders [48,49].

The existence of the double burden of malnutrition in LMIC, described by a WHO consultation group, is observed in our study population [50]. The higher prevalence of underweight among men compared to women is in line with findings from other studies in urban Africa [51,52,53]. The patterns of the double burden of malnutrition that was observed may be influenced by factors such as economic growth, social change, and urbanization occurring in the continent. For example, food insecurity has always been associated with rural communities in Africa [54], but, recently, urbanization has caused a major shift in poverty from individuals migrating from rural to urban areas, and consequently shifting the issue of underweight to urban cities [55]. Many health-related policies in Africa put emphasis on reducing underweight and infectious diseases, overlooking the rapidly growing prevalence of conditions that are related to overnutrition. For instance, the United Nations Sustainable Development Goals have called on countries to develop policies that deal with issues of poverty, hunger, and health [56]. However, these goals are more focused on underweight populations, and may not be adequate for overweight and obesity related conditions.

In the current study, the prevalence of normal-weight central obesity was high, ranging from 16% to 24% using different measures of abdominal obesity. Despite the strong association between obesity and non-communicable diseases, the distribution of body fat should also be considered to be important when determining this risk. In fact, central obesity has been recognized as an independent risk factor for cardio-metabolic diseases and a better predictor of cardiovascular risk than overall obesity [57]. This risk has also been observed among individuals with normal weight and central obesity [58,59]. Therefore, our findings of a high proportion of normal-weight central obesity are concerning and call for further investigation on its impact on mortality among adults.

To our knowledge, our study was the first study assessing overweight and obesity among adults to be conducted in Bissau, and it provides a much-needed baseline for the evaluation of trends and interventions in Guinea-Bissau, even though it is only assessed the urban population of Bissau. Nevertheless, some limitations need to be discussed. Participants without data on anthropometric measures were excluded from the current analysis. However, the proportion of participants with missing data is low, reducing the expected impact of their exclusion on the validity of our results. Weight was measured to the nearest kg since we used the same analogic scales that were available in the main hospital: Hospital Nacional Simão Mendes, which did not have 100g intervals; therefore, despite the training of the evaluators, we cannot exclude an overestimation of the weight among participants being perceived as overweight or obese and an underestimation among those perceived as underweight. The precision of the estimates obtained for some of the subgroups who were considered in the stratified analyses was limited due to the small sample size of these subgroups, particularly among men, though this does not apply to the overall estimates. Finally, the sampling procedure used in this survey was complex and included random sampling in the selection of the PSU and households, but not for the selection of participants within the household. This may have contributed to an oversampling of women and older participants, who are more likely to be at home. However, a weight correction based on the expected age and sex distribution of the population in Bissau was used to lessen the impact of these disproportions in the mean and prevalence estimates obtained in this study.

The findings of the current survey show that overweight and obesity is already a major public health issue in urban Guinea-Bissau, and increasing trends may be expected, moving towards levels seen in more developed countries. Therefore, cost-effective strategies that can be implemented at the population and individual level are needed to slow down the growing prevalence of overweight and obesity in Bissau. The effective development of these strategies needs to focus on appropriate, culturally acceptable educational and health promoting programmes that will contribute to decreasing the burden of non-communicable diseases and that will ultimately add up to sustainable livelihoods and the eradication of poverty in the country.

## Figures and Tables

**Table 1:** Sociodemographic characteristics of the participants.

	Women (*n* = 572)			Men (*n* = 343)		
Sociodemographic Characteristics	*n*	Unweighted, %	Weighted, %	*n*	Unweighted, %	Weighted, %
Age, years						
18–24	146	24.5	30.5	87	25.3	27.6
25–34	193	35.1	30.7	120	34.7	34.9
35–44	117	20.3	21.4	67	20.1	18.6
45–54	61	10.5	10.6	35	10.0	10.9
55–69	55	9.7	6.7	34	9.9	8.0
Education, years						
No School	103	18.0	19.8	19	5.7	5.6
1–6	161	27.8	26.8	76	21.9	21.6
7–12	192	34.6	36.2	141	40.9	42.3
>12	107	18.5	15.5	106	31.3	29.8
Not reported	9	1.1	1.6	1	0.3	0.6
Income, USD/year						
No income	241	42.2	43.7	120	34.9	32.9
1 ≤ 568	47	7.6	11.9	13	3.7	5.9
570 ≤ 1898	72	12.4	12.5	65	19.0	17.4
≥1900	25	4.2	2.5	30	8.5	9.7
Not reported	187	33.7	29.3	115	33.8	34.1

**Table 2:** Mean body mass index and waist circumference, and prevalence of body mass index categories and abdominal obesity, according to gender, among adults aged 18–69 years living in Bissau.

	Women		Men	
	Mean or %	95% CI	Mean or %	95% CI
BMI (kg/m^2^), mean	25.5	24.5–26.6	22.8	22.0–23.5
BMI category, %				
Underweight (<18.5 kg/m^2^)	10.2	6.9–15.0	13.3	8.1–21.0
Normal (18.5–24.9 kg/m^2^)	41.3	34.0–49.0	61.8	53.2–69.8
Overweight (25.0–29.9 kg/m^2^)	27.8	22.7–33.5	19.3	14.1–25.8
Obesity (≥30.0 kg/m^2^)	20.6	14.8–27.9	5.5	2.9–10.3
Overweight and obesity (≥25.0 kg/m^2^)	48.7	40.2–57.4	25.0	18.5–32.9
Waist circumference (cm), mean	84.0	81.3–86.6	80.1	78.0–82.2
Abdominal obesity *, %	55.8	46.9–64.5	13.9	9.2–20.6

BMI—body mass index. * Defined as waist circumference greater than 80 and 94 cm in women and men, respectively.

**Table 3:** Prevalence of overweight and obesity and mean waist circumference among women and men according to age, education, and income in adults aged 18 to 69 years living in Bissau.

	Women				Men			
	Overweight and Obesity		Waist Circumference		Overweight and Obesity		Waist Circumference	
	%	95% CI	Mean	95% CI	%	95% CI	Mean	95% CI
All participants	48.7	40.2–57.4	84.2	81.5–86.8	25.0	18.5–32.9	80.1	78.0–82.2
Age, years								
18–24	29.3	19.1–42.1	76.6	73.2–80.1	6.3	2.6–14.3	71.2	69.4–73.1
25–34	50.0	38.8–61.2	81.8	79.3–84.4	24.3	14.0–38.7	79.4	76.6–82.2
35–44	64.7	47.2–79.0	91.8	87.5–96.0	33.5	21.4–48.4	85.7	82.1–89.3
45–54	70.0	52.6–83.1	91.3	85.6–96.5	37.6	22.9–54.9	89.4	85.3–93.5
55–69	46.7	21.6–73.6	93.1	88.5–97.6	56.0	35.3–74.7	89.0	83.3–94.7
Education, years								
No School	47.8	31.0–65.0	87.5	82.7–92.2	25.4	8.7–54.8	84.1	79.2–89.0
1–6	43.2	33.2–53.7	82.2	78.7–85.8	14.6	7.4–26.8	77.5	74.3–80.7
7–12	53.4	42.4–64.0	84.6	81.2–88.0	24.0	16.0–34.3	79.1	76.4–81.8
>12	50.5	39.6–61.4	82.8	79.9–85.7	34.5	24.7–45.8	82.9	80.3–85.5
Income, USD/year								
No income	43.2	30.4–56.9	83.4	79.0–87.8	18.6	11.4–29.0	76.6	74.4–78.9
1–569	67.0	52.4–79.0	85.7	81.9–89.4	11.9	2.5–41.4	79.1	68.0–90.3
570–1899	58.9	46.6–70.0	85.0	82.0–87.9	37.6	23.8–53.8	82.3	79.5–85.1
≥1900	69.5	45.7–86.0	86.0	80.7–91.2	35.0	14.0–64.1	89.0	85.0–92.9

**Table 4:** Crude and adjusted odds ratios for the association between sociodemographic characteristics and overweight and obesity, compared with normal weight.

	Overweight and Obesity			
	Crude OR	95% CI	Adjusted OR *	95% CI
Sex				
Male	1.00	Ref	1.00	Ref
Female	2.84	1.88–4.31	4.20	1.71–4.13
Age, years				
18–24	1.00	Ref	1.00	Ref
25–34	2.54	1.52–4.25	2.68	1.71–4.13
35–44	4.50	3.07–6.59	5.80	3.24–10.36
45–54	5.14	2.96–8.91	7.09	3.83–13.11
55–69	4.92	2.13–11.37	7.95	3.00–21.13
Education, years				
No School	1.00	Ref	1.00	Ref
1–6	0.57	0.27–1.22	1.47	0.63–3.41
7–12	0.79	0.41–1.53	2.59	1.16–5.81
>12	0.89	0.41–1.91	3.00	1.23–7.27
Income, USD/year				
No income	1.00	Ref	1.00	Ref
1–569	1.93	0.94–4.01	1.47	0.74–2.93
570–1899	1.80	1.09–2.98	1.59	0.89–2.81
≥1900	1.50	0.56–4.01	1.21	0.49–2.96

* Odds ratios from models including sex, age group, education, and income as independent variables.

**Table 5:** Prevalence of normal-weight central obesity among individuals with normal BMI (18.5–24.99 kg/m^2^) according to waist circumference, waist-to-hip ratio, and waist-to-height ratio by gender and age group.

	Age Groups (Years)	Women		Men	
		%	95% CI	%	95% CI
Waist circumference	Overall	32.7	21.0–47.0	5.9	2.3–14.5
	18–24	25.8	14.0–42.6	–	–
	25–34	27.7	16.6–42.6	5.5	1.2–20.1
	35–44	27.6	9.7–57.5	7.0	1.4–27.9
	45–54	42.6	16.1–74.3	16.7	4.5–46.3
	55–69	85.0	54.1–96.5	21.0	5.1–55.9
Waist-to-hip ratio	Overall	25.5	17.4–35.9	17.7	11.6–26.1
	18–24	23.3	12.4–39.4	5.5	1.2–21.8
	25–34	14.7	7.8–25.9	11.0	44.7–24.7
	35–44	15.2	4.6–40.0	24.9	10.1–49.3
	45–54	41.0	15.2–72.8	40.4	21.4–62.7
	55–69	83.0	50.1–95.9	64.8	33.5–87.1
Waist-to-height ratio	Overall	30.4	19.5–44.2	20.0	12.6–30.15
	18–24	21.6	11.3–37.4	0.2	0.0–1.7
	25–34	28.1	16.9–42.9	11.4	4.6–25.5
	35–44	20.9	7.8–45.4	28.7	12.5–53.0
	45–54	42.6	16.0–74.3	63.8	31.2–87.3
	55–69	89.6	60.0–98.0	79.2	49.7–93.6

## Data Availability

The data presented in this study are available on request from the corresponding author. The data are not publicly available due to lack of permission from the participants.
